# Unveiling the complexity: coexistence of rheumatic heart disease and pulmonary arteriovenous malformation—a unique case report

**DOI:** 10.1093/ehjcr/ytae239

**Published:** 2024-05-09

**Authors:** Riyaz Charaniya, Jayal Shah, Apoorva M

**Affiliations:** Department of cardiology, U. N. Mehta Institute of Cardiology and Research Centre (UNMICRC), Civil Hospital Campus, Asarwa, Ahmedabad 380016, Gujarat, India; Department of cardiology, U. N. Mehta Institute of Cardiology and Research Centre (UNMICRC), Civil Hospital Campus, Asarwa, Ahmedabad 380016, Gujarat, India; Department of cardiology, U. N. Mehta Institute of Cardiology and Research Centre (UNMICRC), Civil Hospital Campus, Asarwa, Ahmedabad 380016, Gujarat, India

**Keywords:** Case report, Embolotherapy, Pulmonary arteriovenous malformation, Rheumatic mitral stenosis, Vascular plug

## Abstract

**Background:**

The coexistence of rheumatic heart disease (RHD) and pulmonary arteriovenous malformation (PAVM) is a rare clinical scenario that poses diagnostic and therapeutic challenges. This case report explores the clinical presentation, diagnostic journey, and multidisciplinary management of a patient presenting with both conditions.

**Case summary:**

A 47-year-old female with a history of RHD presented with symptoms of dyspnoea on exertion and cyanosis, suggestive of both cardiac involvement and pulmonary involvement. Subsequent investigations involving imaging, echocardiography, and invasive pulmonary angiography revealed the coexistence of RHD and multiple PAVM in the patient’s left lower lobe of the lung. The patient underwent a tailored treatment plan, initially involving percutaneous mitral balloon valvuloplasty for RHD, followed by a staged procedure of transcatheter PAVM closure with Amplatzer™ Vascular Plug II performed 1 month later. Her saturation normalized following the intervention. The patient’s progress was monitored closely, with adjustments made to the treatment plan based on evolving clinical scenarios. The patient remained well in short-term follow-up.

**Discussion:**

This case highlights the complexity of managing patients having two diverse conditions RHD and PAVM coexisting together, thus emphasizing the importance of a multidisciplinary approach. The unique intersection of cardiac and pulmonary pathologies necessitates careful consideration of diagnostic nuances and tailored treatment strategies. Lessons learned from this case offer valuable insights for clinicians encountering similar scenarios and underscore the significance of individualized, patient-centred care in optimizing outcomes for those with dual pathologies.

**Learning points**

- This case highlights the *diagnostic challenges* associated with the coexistence of rheumatic heart disease and pulmonary arteriovenous malformation, emphasizing the need for meticulous diagnostic evaluation
- *Patient-centred* decision-making and long-term *prognostic considerations* are crucial for improved patient care.
- This case underscores the *educational value* of exploring rare coexisting pathologies, emphasizing the importance of continuous learning for healthcare professionals.

## Introduction

Rheumatic heart disease (RHD), a sequela of rheumatic fever, primarily affects the heart valves and can lead to significant morbidity and mortality.^1^ On the other hand, pulmonary arteriovenous malformation (PAVM) is characterized by abnormal connections between pulmonary arteries and veins, disrupting normal oxygenation.^2^ Coexistence of PAVM along with severe rheumatic mitral stenosis represents a rare and intriguing clinical scenario that challenges conventional diagnostic and therapeutic paradigms. This case report aims to explore the diagnostic nuances, treatment considerations, and collaborative approaches required when confronted with the uncommon coexistence of RHD and PAVM. To date, only 15 cases of PAVM coexisting with RHD have been reported, among which only five were treated simultaneously by surgery.

## Summary figure

**Table:** Timeline of critical events

2022	Patient first diagnosed with RHD
2023	
Day 1	Patient presented with dyspnoea on exertion (Class III) and cyanosis. Clinical examination and baseline investigations established a diagnosis of RHD with severe mitral stenosis and coexisting pulmonary AV malformation.
Day 8	After initial stabilization, patient was taken up for BMV procedure. After procedure, patient developed a TIA episode.
Day 10	Patient was hence stabilized and discharged and advised to follow-up after 1 month for staged procedure of transcatheter AVM embolization.
Re-admitted 1 month later	
Day 1	Patient admitted for staged procedure
Day 2	Successful AVM embolization done
Day 4	Patient had uneventful post-procedure recovery. Patient discharged and advised to follow-up in OPD after 1 month.

## Case presentation

**Figure 1:**
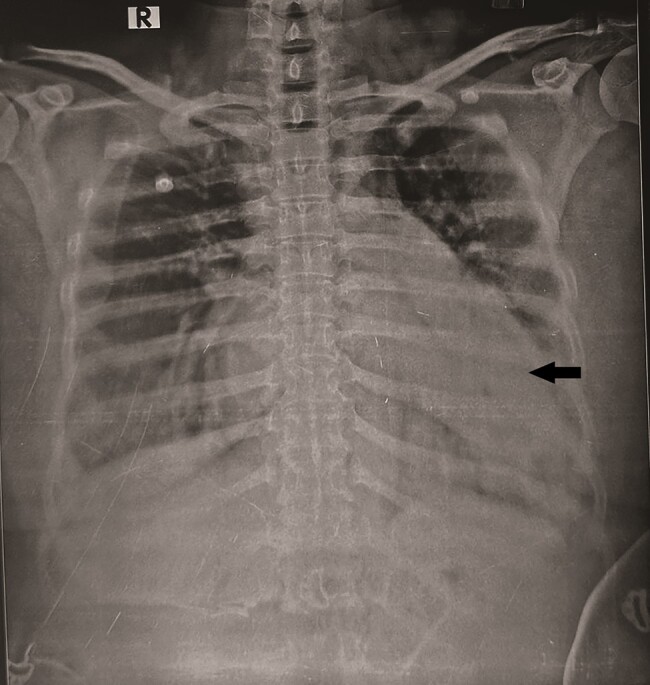
Chest X-ray showing mild para-hilar congestion and diffuse opacification in the left lower lobe of the lung (arrow).

**Figure 2:**
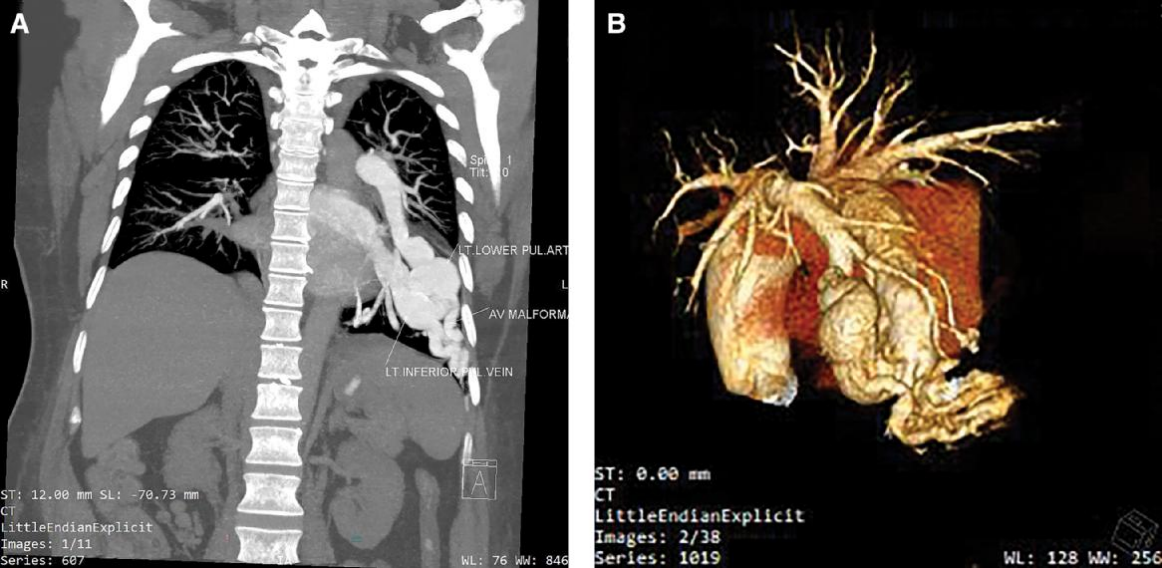
(*A* and *B*) Multiple pulmonary arteriovenous malformation in the left lower lobe of the lung, involving the left lower lobe pulmonary artery.

A 47-year-old female with RHD presented with complaints of dyspnoea on exertion (Class III) for 7 days. On presentation, the patient was hypoxic with SpO2 of 85% at room air and a respiratory rate of 22 breaths/min. Oxygen supplementation showed no clinical improvement. Physical examination showed clubbing and cyanosis, suspecting right to left shunting of blood. The first heart sound was loud; the pulmonary component of the second heart sound was accentuated. There was a mid-diastolic murmur at the apex with pre-systolic accentuation. Chest X-ray showed diffuse opacification in the left lower lobe of the lung with mild para-hilar congestion (*Figure 1*). Echocardiography was done, which showed thickened mitral valve and severe mitral stenosis [mitral valve area (MVA) was 1.1 cm^2^ by 2D planimetry] with mild mitral regurgitation. The left atrium was dilated (size = 54 × 70 mm) with indexed left atrial volume of 105.72 mL/m^2^ and moderate tricuspid regurgitation with mild pulmonary artery hypertension (right ventricular systolic pressure = 39 mmHg) was present. Computed tomography (CT) pulmonary angiography was done, which revealed multiple PAVMs in the left lower lobe of the lung, involving the left lower lobe pulmonary artery (*Figure 2A* and *B*).

**Figure 3:**
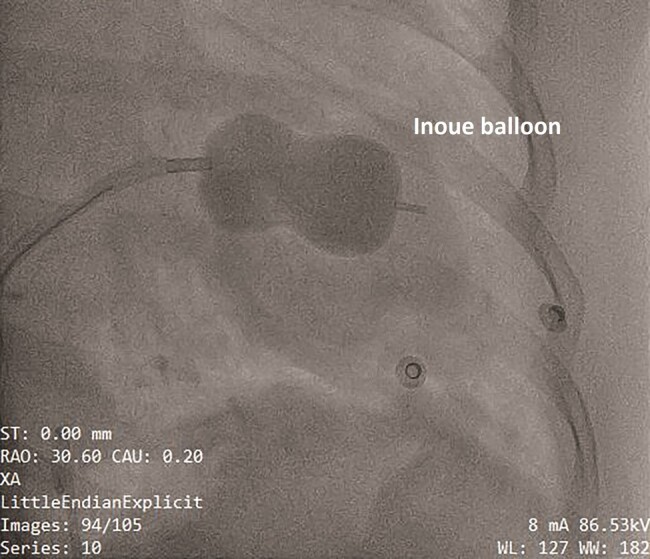
Balloon mitral valvuloplasty using Inoue balloon.

A complex intervention was planned consisting of balloon mitral valvuloplasty (BMV) and transcatheter embolotherapy of AVM by vascular plug. During the pre-operative cardiac catheterization, the basal mean pulmonary artery pressure was 18 mmHg, central aortic pressure was 101/82 mmHg (mean of 78 mmHg), and transmitral gradient (TMG) was 18 mmHg. The patient underwent BMV using 26 mm Inoue balloon (*Figure 3*). The procedure was successful with MVA improving to 1.9 cm^2^, and TMG was 2 mmHg. The patient had one episode of transient ischaemic attack (TIA) immediately after the procedure. The patient recovered from TIA within 30 min with normal CT brain imaging. Hence, the embolization of AVM was planned as a staged procedure after 1 month.

**Figure 4:**
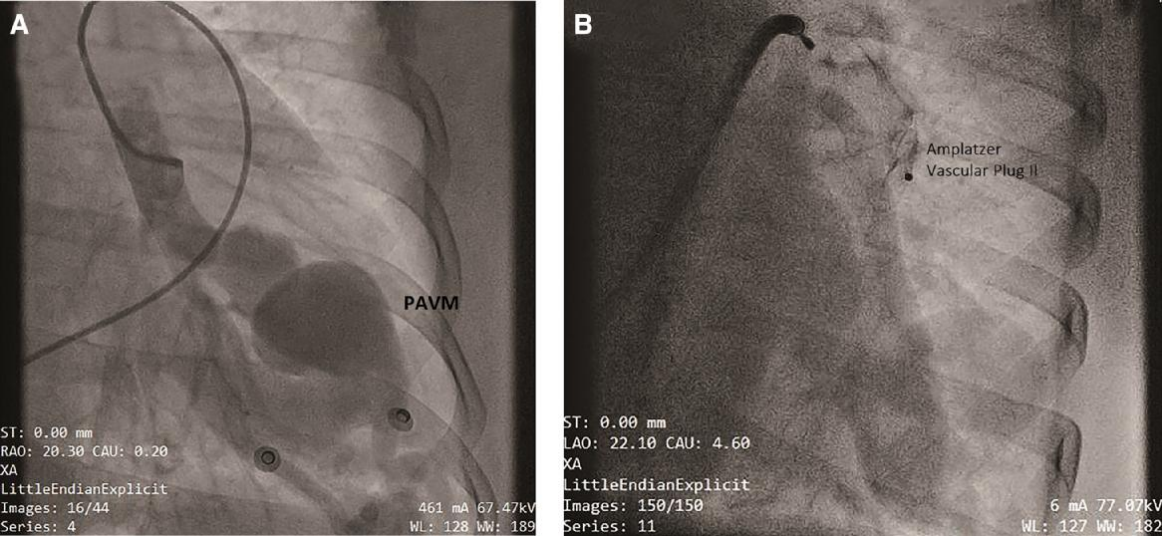
(*A* and *B*) Pulmonary arteriovenous malformation on pulmonary angiogram, occluded using Amplatzer™ Vascular Plug II.

**Figure 5:**
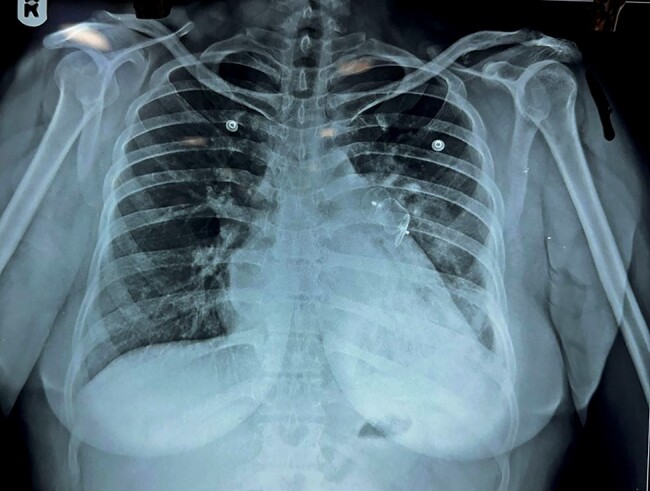
Post-intervention chest X-ray showing the vascular plug *in situ*.

The patient was re-admitted after 1 month for the staged procedure. Pulmonary angiogram done via transfemoral vein access showed large and tortuous feeding artery (diameter = 14 mm) from the left lower branch of the pulmonary artery draining into the left lower pulmonary vein (*Figure 4A*). The fistula was entered with 5F Judkins right coronary artery catheter and 0.035 Terumo guidewire. An 8F long sheath was placed in the fistula over an Amplatz 0.035 super stiff wire. A 20 mm (30–50% larger than target vessel diameter) Amplatzer™ Vascular Plug II was selected as it had nitinol wire mesh ensuring rapid embolization and good on-table occlusion. The device was deployed in the fistula at the level of the left lower pulmonary artery branching under angiographic guidance (*Figure 4B*). Post-occlusion angiogram showed no significant feeding vessel draining to the left atrium. The basal oxygen saturation in the arterial blood gas improved from 87% to 98%. Post-intervention chest X-ray showed vascular plug in place with the clearing of lung opacification in the left lower zone (*Figure 5*). The post-intervention course was uneventful. On short-term follow-up, the patient remained well and her oxygen saturation was 98% on room air.

## Discussion

Pulmonary arteriovenous malformation has an incidence of two to three per 100 000 population.^3^ Sloan and Cooley^4^ in their 15 000 consecutive autopsies found only three cases of PAVM. Incidence of 4.3 cases per year, 194 cases of PAVM, was reported by Mayo Clinic over 45 years. The male-to-female ratio varies from 1:1.5 to 1.8.^5^

Majority of PAVMs (80%) are congenital and unilateral. Hereditary haemorrhagic telangiectasia is the most common congenital cause.^6^ Majority of cases are diagnosed in the first three decades of life. The acquired causes are long-standing hepatic cirrhosis, infections (actinomycosis and schistosomiasis), metastatic lesions, amyloidosis, chest trauma, and previous cardiac surgeries (Glenn operation). It classically presents with dyspnoea, cyanosis, and haemoptysis. Platypnoea and orthodeoxia are also commonly encountered. Transient ischaemic attack occurs in up to 57% of patients with PAVM and symptomatic cerebrovascular accident in 18%.^7^

Pulmonary arteriovenous malformation has been categorized based on feeding segmental artery as simple, complex, and diffuse. The lower lobes are most commonly involved with the left more involved than the right side, although any part of the lung can be affected. The reasons behind the apparent prevalence in the left lower lobe may be due to its embryonic development and exposure to specific hemodynamic conditions that make it more susceptible.^8^ Bilateral lung affection is reported in 8–20% of cases.^9,10^

Contrast echocardiography is usually done to detect the shunt. It does not provide quantitative or anatomic detail of the shunt. It involves the injection of agitated saline or dye into a peripheral vein, and in case of PAVM, there is a delay of three to eight cardiac cycles before the bubbles are visualized in the left atrium.^11^ The vascular anatomy of PAVM can be studied using contrast-enhanced CT. Computed tomography scan was significantly better than conventional angiography in detecting a PAVM (98% vs. 60%).^12^ Computed tomography imaging is essential for optimizing pre-procedural planning of transcatheter-based valvular interventions too, such as assessment of mitral valve area, calcification, and sub-valvular pathology.^13^ Pulmonary angiography, although an invasive tool, remains the gold standard for confirming the diagnosis of PAVM.

Patients with untreated PAVM have high morbidity (50%), compared with patients who received treatment (3%). The goal of treating PAVM is to prevent complications like stroke, abscess, and haemoptysis. Traditionally, surgical resection of the lung was done for the treatment of PAVM, which is now reserved for patients not amenable to catheter embolization therapy.^14^ The predominant treatment for PAVM in recent years has been percutaneous embolization of coils or nitinol occluding devices. Clinicians should consider the type, number of PAVMs, the largest diameter of the feeding artery, and clinical symptoms when managing patients with PAVMs.^15^ Pulmonary arteriovenous malformations with a feeding vessel diameter of 3 mm or larger are embolized.^16–18^ Embolization therapy has become the procedure of choice with success rates greater than 93% and with very few serious complications and minimal loss of pulmonary parenchyma.^19^

The presence of severe rheumatic mitral stenosis can augment the size of PAVM slowly due to increased pulmonary flow, chronic hypoxemia, and associated pulmonary hypertension, contributing to pulmonary vascular remodelling.^20^ The combined impact of RHD and PAVM may contribute to the development or exacerbation of heart failure, increase the risk of thromboembolic events, and increase the susceptibility to endocarditis.

A short-term post-procedure follow-up (1–2 weeks) is recommended for clinical assessment, imaging, relevant laboratory tests to assess for potential complications, and medication adjustment.

## Conclusion

The unusual combination of an anomaly like PAVM with RHD makes it a challenging entity. The classic triad of dyspnoea on exertion, cyanosis, and clubbing should alert the physician to the possibility of PAVM. Detailed clinical and radiological evaluation by a multidisciplinary team is essential for diagnosis and planning management. Transcatheter embolization of the AVM along with appropriate valvular intervention is the definitive management for better outcome.

## Data Availability

The data underlying this article cannot be shared publicly due to the privacy of individuals who participated in the study. The data will be shared on reasonable request to the corresponding author.

## Lead author biography

Jayal Shah, MD, DM(CARD), FSCAI (USA), FESC, FAPSIC, is an interventional cardiologist and a professor in Cardiology at U. N. Mehta Institute of Cardiology and Research Centre, B.J. Medical College, Civil Hospital, Ahmedabad, an international associate member of American College of Cardiology and ESC Council of Cardio-Oncology and ESC Council of Cardiovascular Genomics, and PG teacher for DM Cardiology at Gujarat University.

**Consent:** The authors confirm that written consent for submission and publication of this case report including images and associated text has been obtained from the patient in line with the COPE guidance.

**Funding:** None declared.
